# The Diversity and Function of Soil Bacteria and Fungi Under Altered Nitrogen and Rainfall Patterns in a Temperate Steppe

**DOI:** 10.3389/fmicb.2022.906818

**Published:** 2022-06-28

**Authors:** Yang Yu, Lu Liu, Jianing Zhao, Shuchen Wang, Yijun Zhou, Chunwang Xiao

**Affiliations:** ^1^Key Laboratory of Ecology and Environment in Minority Areas, Minzu University of China, National Ethnic Affairs Commission, Beijing, China; ^2^College of Life and Environmental Sciences, Minzu University of China, Beijing, China

**Keywords:** nitrogen addition, rainfall addition, rainfall reduction, bacterial diversity, fungal diversity, functional prediction, temperate steppe

## Abstract

The response of soil microorganisms to altered nitrogen (N) and rainfall patterns plays an important role in understanding ecosystem carbon and nitrogen cycling processes under global change. Previous studies have separately focused on the effects of N addition and rainfall on soil microbial diversity and community composition. However, the combined and interactive impact of N addition and rainfall on soil microbial diversity and function mediated by plant and soil processes have been poorly investigated for grassland ecosystems. Here, we conducted a field experiment with simulated N addition (N addition: 10 g N m^–2^yr^–1^) and altered rainfall pattern [control, rainfall reduction (compared to control –50%); rainfall addition (compared to control + 50%)] to study their interactive effects on soil microbial diversity and function in a temperate steppe of Inner Mongolia. Our results showed that N addition and rainfall addition significantly increased soil bacterial diversity, and the bacterial diversity was positively correlated with soil microbial biomass nitrogen, inorganic nitrogen, and *Stipa krylovii* root exudate C:N ratio, *Allium polyrhizum* root exudate C and N, and *A. polyrhizum* root exudate C:N ratio. N addition and rainfall reduction significantly reduced fungal diversity, which correlated closely with soil microbial biomass carbon and the C:N ratio of *A. polyrhizum* root exudates. Bacteria were mainly eutrophic r-strategists, and the responses of bacterial function guilds to the interaction between N addition and rainfall pattern were not significant. However, the arbuscular mycorrhizal fungi (AMF), in the functional classification of fungi, were significantly reduced under the condition of N addition and rainfall reduction, and the absolute abundance of the phylum Glomeromycota increased under rainfall addition, suggesting that AMFs are sensitive to altered N and rainfall patterns over short timescales (1 year). Collectively, our results have important implications for understanding the plant–soil–microbe system of grasslands under climate change.

## Introduction

Due to the unreasonable use of resources and the burning of fossil fuels, atmospheric nitrogen (N) deposition has increased by 2–7 fold compared to pre-industrial revolution levels (Galloway et al., 2004). N deposition increased soil nitrogen content, which can affect the growth of plants, quality of grassland forage, and nutrient cycling within ecosystems (Waldrop et al., 2004; Turner and Henry, 2010). Meanwhile, due to natural factors and human activities, global rainfall patterns have also changed at different latitudes (Allen et al., 2018). Previous studies have shown water availability is an important factor limiting the productivity of arid and semi-arid grasslands (Füzy et al., 2008); furthermore, the grassland of Inner Mongolia is an area that is more sensitive to rainfall. Therefore, a better understanding of the impact of climate change on the grassland ecosystem could be gained from studying the interaction between N addition and rainfall.

As decomposers, the diversity, composition, and function of soil microorganisms correlate closely with the biogeochemical cycles and homeostasis of ecosystems (Bardgett and Putten, 2014; Fierer, 2017; Tian et al., 2021). N deposition enhanced the photosynthetic capacity of plants (Wang et al., 2012), leading to an increase in plant biomass and the quantity of plant litter, and then acting as a sufficient nutrient source for soil microorganisms and affecting microbial diversity, community structure, and function. Previous studies have shown that N deposition increased the competition among plant species and decreased plant species diversity, thus affecting the food source and living microenvironments of soil microorganisms and resulting in the variation of soil microbial diversity (Fierer and Jackson, 2006; Fierer et al., 2007; Rousk et al., 2010). The variation of rainfall is an important factor affecting global climate change (Liu et al., 2013; Marvel and Bonfils, 2013). Rainfall addition increases soil moisture and affects the relative abundance of soil fungi and Gram-negative bacteria, resulting in altered bacteria: fungi ratios, which further impact the bacteria and fungi that participate in soil carbon and nitrogen cycling (Williams and Rice, 2007). Also, Jie et al. (2011) have documented that changes in rainfall patterns influence soil N availability. In brief, N and water are two important factors that limit soil microbial activity in the grasslands of northern China (Zhang et al., 2015). A previous study showed that precipitation was an important environmental factor affecting soil microbial carbon utilization profiles based on BIOLOG carbon substrate utilization under N addition (Sun et al., 2015). However, the functional classification of microorganisms involved in carbon cycling is still insufficient. Plants secrete a variety of root exudates during the process of growth, and these exudates act as the interaction medium between plants and microorganisms. The composition of root exudates is known to affect the soil microbial diversity and function (Baudoin et al., 2003). However, the mechanism by which the C:N ratio of root exudates affects soil microbe is still unclear. Understanding the C:N ratio of root exudates lays a foundation for explaining how plant–soil–microbial interactions play significant roles in ecosystem processes, such as carbon and nitrogen nutrient cycling.

Liang et al. (2020) have reported long-term fertilization significantly changed the composition of soil microbial community, which in turn affects the functional characteristics of the community (Fukami, 2015). It was reported that Proteobacteria, Acidobacteria, and Actinobacteria dominated the bacterial communities in the typical steppe of northern China, and that the fungal community was mainly composed of Ascomycota, Basidiomycota, and Glomeromycota (Yu et al., 2021a). Actinobacteria, Proteobacteria, and Bacteroidetes are classified as r-strategists, mainly decomposing labile C, while most Acidobacteria are classified as K-strategists and decompose recalcitrant C (Sun et al., 2021). However, it is not clear whether N addition, rainfall, and their interaction are dominated by r-strategist bacteria and accelerated soil carbon and nitrogen cycling. At present, the effects of fertilization on fungal functional groups mainly focused on arbuscular mycorrhizal fungi (AMF). AMF play important roles in plant growth, by providing multiple nutrients and assisting the host plants to resist pathogen invasion. Chen et al. (2017) have found that fertilization has decreased AMF α-diversity and altered community composition and phylogenetic patterns, suggesting that environmental factors are important driving forces of AMF community assembly. Nevertheless, the effect of interaction between N addition and rainfall on fungal functional classification is still unknown.

In order to understand how microbial diversity and function are affected by altering N and rainfall patterns, we conducted a short-term (1 year) field N addition and rainfall interaction experiment in a temperate steppe of Inner Mongolia. The main objectives of this study were (1) to assess the effects of N addition, rainfall, and their interaction on soil microbial diversity and community composition, and to confirm whether the community composition mainly belongs to r-strategists, and (2) to examine the functions of soil microorganisms in response to N addition, rainfall, and their interaction. Since bacteria are more sensitive to changes in soil moisture than fungi (Gordon et al., 2008), we hypothesized that N and rainfall interaction would (i) increase soil bacterial diversity, while not affecting fungal diversity; (ii) may increase the soil inorganic nitrogen content and soil water content and create sufficient food sources that, in turn, will increase bacterial diversity and affect bacterial community composition, mainly belonging to r-strategies; and (iii) the increased soil nutrient input will accelerate soil bacterial cycling of soil carbon and nitrogen, and enhance the functional classes of microorganisms involved in soil carbon and nitrogen cycling. Thus, the interaction between N and rainfall may increase the relative abundance of the AMF.

## Materials and Methods

### Site Description

We conducted a field experiment in a temperate steppe at the Hulun Lake Reserve in Inner Mongolia (113.21°E, 48.75°N), China. This site is located in a temperate zone with a mean annual temperature of –0.4°C and mean annual precipitation of 283 mm, respectively. The mean temperature was 1.8°C, and the precipitation during the growing season was 169 mm in 2018. The soil is classified as Calcic Luvisols (FAO, 1957). The vegetation is dominated by perennial species, primarily *Stipa krylovii* and *Allium polyrhizum*.

### Experimental Design

The experimental design has been described in detail by Li C. et al. (2021). In brief, the experiment used a factorial, nested design with rainfall treatment [control, reduction (–50%), and addition (+ 50%)] and nitrogen (N) treatments (control and addition). Each treatment had five replicate blocks, for a total of thirty 2 m × 2 m plots that were set up on 1 June 2018. The rainfall treatments were covered by transparent rainfall shelters supported on an iron frame roof, the rainfall reduction was collected through a PVC pipe into a barrel, and rainfall addition was applied by respraying the collected rainwater from the bucket onto the treatment area. The N addition rate (in the form of NH_4_NO_3_) was 10 g N m^–2^ yr^–1^, which was applied in June, July, and August.

### Field Sampling

Soil samples were collected on 10 August 2018. Five soil cores (10 cm in diameter) were randomly collected from 0 to 10 cm topsoil and then mixed to create a composite sample for each plot. The composite soil samples were sieved through a 2.0-mm sieve to remove plant debris and roots. The sieved soil samples were then separated into two parts, with one stored at 4°C for testing of physical and chemical properties and one at –80°C for high-throughput sequencing of microorganisms. The aboveground biomass was measured by cutting living plants at ground level within 0.5 m × 0.5 m quadrat. The belowground biomass was estimated by using the soil cores method (Vogt and Persson, 1991). The root exudation of *S. krylovii* and *A. polyrhizum* was collected by using a modified method in August 2018 (Phillips et al., 2008). The methods of collecting root exudates and measuring the carbon and nitrogen of root exudates were described in detail by Li C. et al. (2021).

### Lab Analysis

Soil samples were oven-dried for 48 h at 105°C to obtain soil moisture content. Ten grams of air-dried soil was used to calculate the soil ammonia nitrogen and nitrate nitrogen with 50 ml of 2 M KCl, shaken for 30 min, and the filtered solution was measured by using a continuous flow analyzer (Futura, Alliance Instruments, Frépillon, France). Soil microbial biomass carbon and nitrogen contents (SMBC and SMBN, respectively) were measured using the fumigation extraction method (Vance et al., 1987). Twenty-five grams of fresh soil was extracted with 100 ml of 0.5 M K_2_SO_4_, and the extracted solutions were analyzed by a TOC analyzer (Analytik, Jena, Germany). Aboveground and belowground biomass samples were oven-dried at 65°C to a constant weight.

### DNA Extraction and Polymerase Chain Reaction Amplification and Sequencing

Soil DNA was extracted from 0.5 g of fresh soil using the PowerSoil expand DNA Isolation Kit (MO BIO) according to the manufacturer’s instructions. Nanodrop Spectrophotometer (Nano-100, Aosheng Instrument Co Ltd.) assays qualified genomic DNA concentration and polymerase chain reaction (PCR) amplification products. The primers 515F (5′-GTGCCAGCMGCCGCGGTAA-3′) and 806R (5′-GGACTACHVGGGTWTCTAAT-3′) were used to amplify the V4 region of bacteria 16S rRNA gene (Caporaso et al., 2012). In order to distinguish the different samples, a barcode sequence was added to the 5′ – end of the reverse primer. The PCR was performed in a 50-μl reaction mixture containing 20 μl of nuclease-free water, 25 μl of 2 × Primer Taq (Takara, RR902A), 1 μl of forward primer (10 mM), 1 μl of reverse primer (10 mM), and 3 μl of g-DNA. PCR cycling conditions were set for 95°C for 5 min, followed by 40 cycles of denaturation at 94°C for 30 s, annealing at 53°C for 30 s, extension at 72°C for 40 s, with a final extension of 72°C for 8 min. For fungi, the primers ITS3 (5′-GCATCGATGAAGAACGCAGC-3′) and ITS4 (5′-TCCTCCGCTTATTGATATGC-3′) (Ihrmark et al., 2012) were used to amplify the ITS2 region (Gardes and Bruns, 2010). The PCR reaction mixture was the same as that used for the bacterial 16S rRNA gene. PCR cycling conditions were set at 95°C for 5 min, followed by 40 cycles of denaturation at 94°C for 30 s, annealing at 58°C for 30 s, extension at 72°C for 40 s, with a final extension of 72°C for 8 min. The PCR products were purified using the Omega kit following the manufacturer’s protocol. The purified PCR products were paired-end sequenced using Illumina HiSeq 2500 platform. We also quantified the absolute abundance of 16S rRNA and ITS genes. The reaction mixture for qPCR included 10 μl of 2 × qPCR mix, 2 μl of g-DNA, 2 μl of 2.5 μM (515F/806R and ITS3/ITS4) primers, and 6 μl of ddH_2_O. PCR cycling conditions were set at 95°C for 5 min, followed by 31 cycles of denaturation at 95°C for 30 s, annealing at 53°C for 15 s (16s rRNA) and 58°C for 15 s (ITS RNA), and extension at 72°C for 30 s. The melt curve was set for temperature from 60 to 95°C, and the curve was checked for every increase of 0.3°C.

### Sequence Data Processing

Raw sequencing data were processed using an in-house Galaxy Pipeline^1^ (Feng et al., 2017). We detected barcodes used Trimmomatic-0.33 to discard the low-quality sequences and obtain high-quality sequences. We use Flash to combine the forward sequences and reverse sequences. Reads were assigned to the same OTU by using the UPARSE method according to the 97% identity threshold. All the analysis is based on the generated OTU table. 16S and ITS OTU representative sequences were taxonomically assigned by using RDP Classifier against the 16S ref. Silva database and ITS Refs database (Zhou et al., 2011). On average, we obtained high-quality sequences with 48,783 16S rRNA sequences and 42,487 ITS sequences per sample across all soil samples. We then resampled 33,371 16S rRNA sequences and 27,731 ITS sequences per sample for further microbial analyses.

### Data Analysis

Based on the resampled OTU table, we calculated the bacterial and fungal α-diversity (Shannon index and Inv-Simpson Index). We measured the influences of N and rainfall patterns on the α-diversity index of bacteria and fungi by using one-way ANOVA analysis. The relative abundance of species was estimated through the data analysis platform (see text footnote 1) (Louca et al., 2016). Pearson correlation analysis was used to evaluate the relationship between soil physicochemical properties, plant root exudation, and bacteria α-diversity by using ggcorplot and ggthemes packages in RStudio (3.6.2). LefSe analysis was used to test the bacterial and fungal biomarker groups for N addition and altered rainfall patterns. LDA (linear discriminant analysis) measured the relative abundance of bacterial and fungal species (Yu et al., 2021a). We used the FAPROTAX database and FUNGuild pipeline to predict the bacterial and fungal functions by data process platform (see text footnote 1) (Yu et al., 2021b). Mantel and partial Mantel tests were used to examine the relationships between the Glomeromycota community composition and environmental variables.

## Results

### Plant Biomass and Soil Chemo-Physical Properties

The results of aboveground biomass, belowground biomass, and root C and N exudation rates and C:N ratios of root exudates, soil moisture, soil nitrate, and ammonium were previously published in Li C. et al. (2021). The SMBC significantly increased under nitrogen (N) addition and N addition and rainfall addition interaction (RA + N), and significantly decreased under rainfall reduction (RR) (Supplementary Table 1). The SMBN significantly increased under N addition, N addition and rainfall reduction interaction (RR + N), and RA + N (Supplementary Table 1). The SMBC:SMBN ratio significantly decreased in response to RR (Supplementary Table 1).

### Factors Influencing Bacterial and Fungal Diversity

**FIGURE 1:**
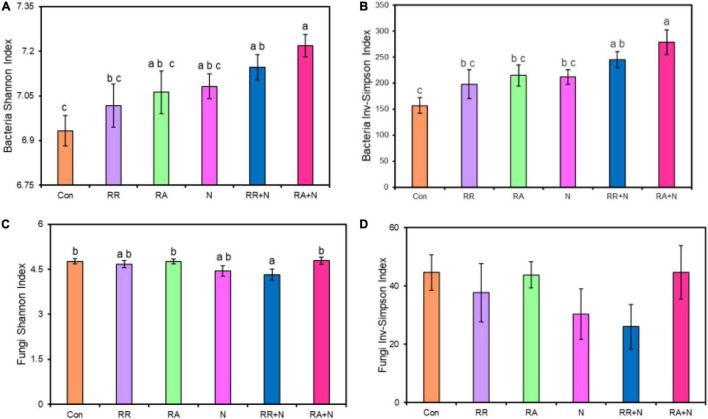
The effects of altered nitrogen addition and rainfall, and their interaction, on bacterial Shannon index **(A)** and Inv-Simpson index **(B)**, fungal Shannon index **(C)** and Inv-Simpson index **(D)** in August 2018. Different treatments: Con: Control; RR: Rainfall reduction; RA: Rainfall addition; N: Nitrogen addition; RR + N: Rainfall reduction and Nitrogen addition; RA + N: Rainfall addition and Nitrogen addition. Values are mean ± SE (*n* = 5). Different lowercase letters indicate significant differences (*p* < 0.05) according to Duncan’s *post hoc* test.

**FIGURE 2:**
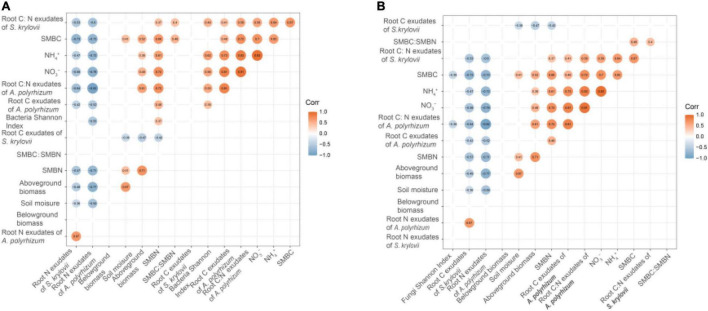
Pearson correlations among bacterial Shannon index **(A)**, fungi Shannon index **(B)**, aboveground biomass, belowground biomass, soil moisture, soil microbial biomass carbon (SMBC), soil microbial biomass nitrogen (SMBN), SMBC:SMBN, NO_3_^–^, NH_4_^+^, root C exudates of *Stipa krylovii*, root N exudates of *S. krylovii*, root C: N exudates of *S. krylovii*, root C exudates of *Allium polyrhizum*, root N exudates of A. polyrhizum, and root C: N exudates of *A. polyrhizum*. The number represents the correlation coefficient. Coefficients that are not correlated have been hidden. Different treatments: Con: Control; RR: Rainfall reduction; RA: Rainfall addition; N: Nitrogen addition; RR + N: Rainfall reduction and Nitrogen addition; RA + N: Rainfall addition and Nitrogen addition.

By themselves, N and rainfall did not significantly affect bacterial and fungal Shannon diversity and Inv-Simpson indices (Figure 1). Bacterial Shannon diversity and Inv-Simpson diversity indices significantly increased under the condition of RA + N (Figures 1A,B), while the fungal Shannon diversity index significantly decreased under rainfall reduction and N addition (RR + N) (Figures 1C,D). Pearson’s correlation analysis indicated that SMBN, inorganic nitrogen (NO_3_^–^ and NH_4_^+^), *S. krylovii* root exudate C:N ratio, and *A. polyrhizum* root exudate C, N, and C:N ratio all significantly affected bacterial Shannon diversity index (Figure 2A). SMBC and *A. polyrhizum* root exudate C:N ratio significantly affected the fungal Shannon diversity index (Figure 2B).

### Bacterial and Fungi Community Composition and Biomarkers

**FIGURE 3:**
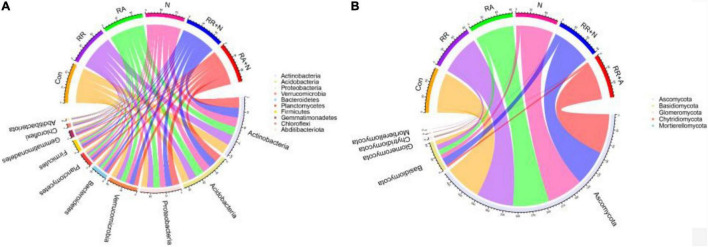
The effects of altered nitrogen addition and rainfall, and their interaction, on the relative abundance of dominant members of the bacterial **(A)** and fungal **(B)** communities in August 2018. Different treatments: Con: Control; RR: Rainfall reduction; RA: Rainfall addition; N: Nitrogen addition; RR + N: Rainfall reduction and Nitrogen addition; RA + N: Rainfall addition and Nitrogen addition.

**FIGURE 4:**
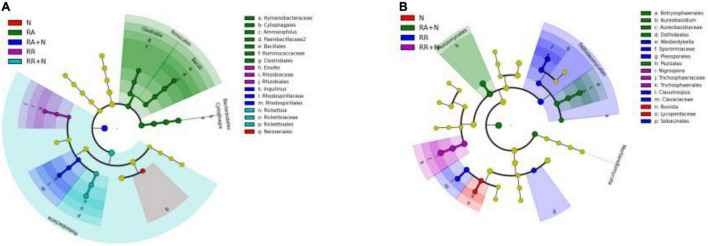
Cladogram indicates the phylogenetic distribution of bacterial lineages (*P* = 0.05, LDA = 2.0) **(A)** and fungal lineages (*P* = 0.05, LDA = 4.0) **(B)** under the treatment of altered nitrogen addition and rainfall patterns. Each circle’s diameter is proportional to the given taxon’s relative abundance (*n* = 5). Different treatments: Con: Control; RR: Rainfall reduction; RA: Rainfall addition; N: Nitrogen addition; RR + N: Rainfall reduction and Nitrogen addition; RA + N: Rainfall addition and Nitrogen addition.

At the phylum level, the bacterial community was dominated by Actinobacteria (mean⋅20%), Acidobacteria (mean 16%), and Proteobacteria (mean 13%) (Figure 3A). The fungal community was mainly constituted of Ascomycota (mean 78%), Basidiomycota (mean 10%), and Glomeromycota (mean 0.70%) (Figure 3B). LefSe showed that the bacterial biomarkers of RR mainly belonged to the phylum Proteobacteria (genus *Ensifer*, family Rhizobiaceae). The biomarkers of RA included the phyla Gemmatimonadetes (class Longimicrobia), Firmicutes (class Clostridia and order Bacillales), Bacteroidetes (order Cytophagales), and class Deltaproteobacteria. The biomarkers of N addition mainly belonged to the phylum Proteobacteria (order Neisseriales). The biomarker bacteria of RR + N mainly belonged to the phylum Proteobacteria, while the biomarkers of RA + N belonged to the phylum Proteobacteria (genus *Inquilinus*, family Rhodospirillaceae, and order Rhodospirillales) (LDA = 2, Figure 4A). The biomarker fungi of RR mainly belonged to the phyla Ascomycota (class Dothideomycetes) and Basidiomycota (genus *Clavulinopsis*, family Clavariaceae, and order Sebacinales). The fungal biomarkers of N treatment mainly belonged to the phylum Basidiomycota (genus *Bovista* and family Lycoperdaceae). The indicator fungi of RR + N treatment mainly belonged to the phylum Ascomycota (genus *Nigrospora*, family Trichosphaeriaceae, and order Trichosphaeriales). The indicator fungi of RA + N treatment belonged to the phylum Ascomycota (class Pezizomycetes, order Botryosphaeriales, genus *Aureobasidium*, family Aureobasidiaceae, and order Dothideales) (LDA = 4, Figure 4B).

### Functional Prediction of Soil Bacteria and Fungi

**FIGURE 5:**
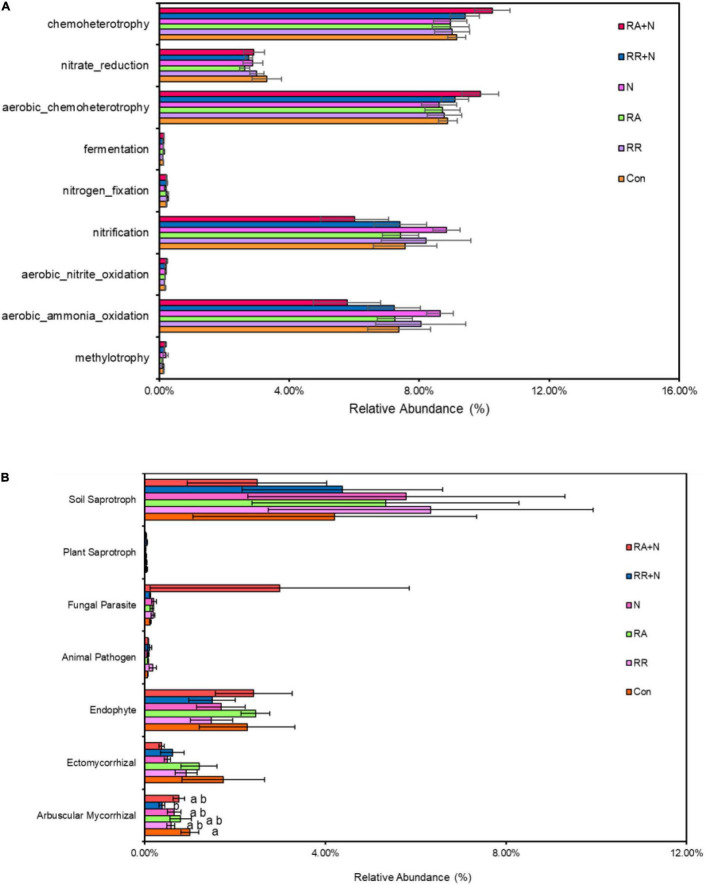
The effects of altered nitrogen addition and rainfall, and their interaction, on the relative abundance of bacterial **(A)** and fungal **(B)** functional groups based on the FAPROTAX tool and FUNGuild pipeline, respectively. Different treatments: Con: Control; RR: Rainfall reduction; RA: Rainfall addition; N: Nitrogen addition; RR + N: Rainfall reduction and Nitrogen addition; RA + N: Rainfall addition and Nitrogen addition. Values are mean ± SE (*n* = 5). Different lowercase letters indicate the significant differences (*p* < 0.05) according to Duncan’s *post hoc* test. Different lowercase letters indicate significant differences (*p* < 0.05) according to Duncan’s *post hoc* test.

**FIGURE 6:**
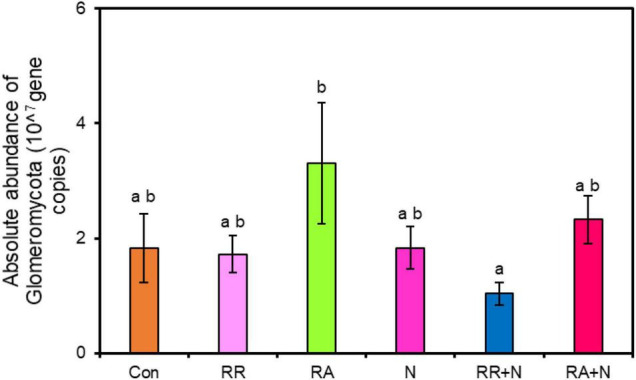
The effects of nitrogen addition and rainfall and their interaction on the absolute abundance of Glomeromycota. Different treatments: Con: Control; RR: Rainfall reduction; RA: Rainfall addition; N: Nitrogen addition; RR + N: Rainfall reduction and Nitrogen addition; RA + N: Rainfall addition and Nitrogen addition. Values are mean ± SE (*n* = 5). Different lowercase letters indicate significant differences (*p* < 0.05) according to Duncan’s *post hoc* test.

There was no significant response by soil bacterial function prediction to N addition and rainfall patterns (Figure 5A). For fungal function prediction, only arbuscular mycorrhizal significantly decreased under the RR + N treatment (Figure 5B). As the arbuscular mycorrhizal (AM) fungi are dominated by the members of the phylum Glomeromycota, within belowground plant–microbe systems (Johnson et al., 2013), by calculating the absolute abundance of this phylum, it was shown that RA treatment significantly increased the absolute abundance of Glomeromycota as compared to RR + N treatment (Figure 6). Mantel test showed that the root C exudation rates of *A. polyrhizum* significantly affected the abundance of Glomeromycota (Table 1).

**TABLE 1:** The phylum Glomermycota relative to OTUs and environmental factors based on Mantel tests.

	R	p
Aboveground biomass	0.0109	0.442
Belowground biomass	–0.0685	0.832
Soil moisture (%)	–0.0035	0.489
SMBC	–0.0643	0.779
SMBN	0.0112	0.407
NO_3_^–^	0.0181	0.278
NH_4_^+^	0.0373	0.251
C exudation rates of *Stipa krylovii*	–0.0459	0.679
N exudation rates of *Stipa krylovii*	–0.0765	0.818
C: N exudation rates of *Stipa krylovii*	–0.0997	0.927
C exudation rates of *Allium polyrhizum*	0.142	0.014*
N exudation rates of *Allium polyrhizum*	–0.057	0.772
C: N exudation rates of *Allium polyrhizum*	0.0095	0.431

* Represent significant differences at p < 0.05.

## Discussion

### Effects of Nitrogen Addition and Rainfall Interaction on Soil Bacterial and Fungal Diversity

Our results showed that nitrogen (N) addition did not affect the diversity of bacteria and fungi, which was consistent with the non-linear response of bacterial diversity to N addition found by Liu et al. (2021a), and supports our first hypothesis. A previous study showed that the diversity of bacteria decreased with N addition when the level of N input was between 16 and 32 g N m^–2^ yr^–1^ (Liu et al., 2021a), while Zhang et al. (2018) found that fungal diversity did not significantly change under N addition alone (< 100 kg N⋅ha^–1^⋅yr^–1^). In our study, N addition was 10 g N m^–2^ yr^–1^, which was below the threshold level required to affect bacteria and fungi, and therefore the α-diversities of bacteria and fungi were not significantly increased by N addition. However, the treatment of RA + N significantly increased the bacterial Shannon diversity index. The interaction between N and rainfall may provide better conditions for bacterial survival, which may be the main reason for the increased bacterial α-diversity under RA + N (Shade et al., 2012). When water is sufficient, N addition has a positive effect on bacterial diversity (Zhang et al., 2015). Intriguingly, our results revealed that N addition alone did not affect fungal diversity and that RR + N decreased fungal diversity, therefore ignoring the factor that rainfall may influence judgment of the effect of N addition on soil fungal diversity. Consistent with our research, Wang H. et al. (2020). showed that rainfall played a key role in influencing the composition of the fungal community under N addition. Therefore, rainfall can alleviate the effects of N deposition on fungal diversity. Previous research found that N addition increased fungal α-diversity, and that the interaction between N and irrigation did not affect fungal diversity (Zhang et al., 2018). In total, soil fungal diversity might be overestimated if variations in moisture under N deposition are ignored (Zhang et al., 2018). Also, previous research, using PLFA technology, provided evidence that N deposition decreased the relative abundance and biomass of bacteria and fungi (Ma et al., 2016; Tian et al., 2017), and that under a high level of N addition (200 kg N ha^–1^ yr^–1^) in the absence of water leaching, the growth of fungi was significantly inhibited (Sun et al., 2015). Therefore, under the interaction of RR + N, the α-diversity of fungi was significantly reduced. Moreover, some studies have indicated that the fungal community was more sensitive than bacteria under N deposition (Herold et al., 2012; Wang J. et al., 2020; Liu et al., 2021b). The long-term response of bacterial and fungal diversity to N addition and rainfall interaction requires further experimental verification.

In grassland ecosystems, soil microbial diversity was influenced by biological and abiotic factors, including soil nutrients, aboveground biomass, and litter quality (Contosta et al., 2015). Using meta-analysis, Wang et al. (2018) showed that decreased soil microbial diversity was associated with the decreased microbial biomass under N deposition. Our results showed that there was a significant correlation between the bacterial diversity and SMBN, ammonia nitrogen, and nitrate nitrogen. A possible explanation for the correlation may be that N addition increases ammonia nitrogen and nitrate nitrogen, stimulating the soil bacteria to uptake inorganic nitrogen and increase bacterial biomass and diversity. Due to the carbon–nitrogen coupling mechanism of bacteria, N input accelerates bacterial carbon uptake and increases bacterial activity, which stimulates the ability of bacteria to degrade litter (Schleuss et al., 2019). This may be a possible explanation for the increase in bacterial diversity under RR + N and RA + N conditions. Our results indicated that the main factors affecting fungal diversity were SMBC and the *A. polyrhizum* root exudate C:N ratio. In general, fungal communities are less affected by the external environment in the short term when they are mycelium rich. However, our study results indicated that soil abiotic and biological factors had significant effects on bacterial and fungal diversity under N, rainfall, and their interaction. It also highlights the impact of climate change on plant–soil–microbe interactions.

### Effects of Nitrogen Addition and Rainfall Interaction on Biomarker Organisms and the Survival Strategies of Soil Bacteria and Fungi

Actinobacteria and Acidobacteria dominated all of the bacterial communities in our study. Based on Pianka’s r/K-selection theory, both Actinobacteria (20%) and Proteobacteria (13%) grow faster and are eutrophic r-strategists (Fierer et al., 2007; Schleuss et al., 2019; Sun et al., 2021), while Acidobacteria (16%) are oligotrophic K-strategists (Sun et al., 2021). The majority of soil bacteria are r-strategists, with a few being K-strategists (Sun et al., 2021). Through Lefse analysis across treatments, we found nitrogen (N) addition mainly increased the relative abundance of the Proteobacteria, which are r-strategists, and therefore, N addition increased the bacterial r-strategist community. A potential explanation for this might be that N addition increased inorganic nitrogen content and stimulated microbial input of nitrogen and labile C (Fontaine et al., 2003; Fierer et al., 2012), resulting in faster microbial growth rates. This is in agreement with Sun et al. (2015) and is consistent with our second hypothesis that copiotrophic groups of bacteria will be mainly observed in a nutrient-rich environment under N addition treatment. Rainfall addition mainly increased the relative abundance of the phyla Gemmatimonadetes, Firmicutes, Bacteroidetes. Gemmatimonadetes, Firmicutes, and Bacteroidetes, all of which are copiotrophic r-strategists (Li H. et al., 2021; Sun et al., 2021). The effect of N and rainfall interaction also increased the relative abundance of the eutrophic bacterial community, which is in agreement with previous research that N and rainfall interaction promoted plant growth, plant litter quantity, dissolved carbon and nitrogen, and root exudate components, therefore accelerated bacterial growth, especially that of copiotrophic r-strategists (Meier and Bowman, 2008; Millard and Singh, 2010). As for fungal community structure, N addition mainly increased the relative abundance of the phylum Basidiomycota, while the effects of N and rainfall interaction increased the relative abundance of Ascomycota and Basidiomycota. These two phyla mainly represent copiotrophic r-strategists and oligotrophic k-strategists, respectively (Yao et al., 2017). The fungal communities in the control soil were also dominated by Ascomycota and Basidiomycota, and N addition and rainfall had little effect on fungi over the short term of our study. In fact, due to the rich mycelium of fungal communities, they are not very sensitive to external environmental stimuli (Berlemont, 2017; Schleuss et al., 2019).

### Effects of Nitrogen and Rainfall Interaction on the Functions of Soil Bacteria and Fungi

In this study, the interaction between N addition and rainfall had no significant effect on the bacteria participating in soil carbon and nitrogen cycling. This was inconsistent with our third hypothesis. Our results suggested that although the bacterial community saw an increased copiotrophic r-strategy groups, there was no obvious effect on the degradation of soil organic matter. The reason for this inconsistency may be that the short timescale (only 1 year) of the treatment did not significantly affect soil physical and chemical properties in our study, and bacteria had functional redundancy that they did not affect the soil carbon and nitrogen cycle. Moreover, soil bacteria can flexibly adapt to environmental changes, leading to bacterial functions not being significantly affected (Shade et al., 2012). Previous studies found that long-term fertilization significantly decreased microbial functional guilds involved in carbon fixation, degradation, N fixation, and mineralization. Because changes in soil functional community structure lead to a decrease in stochastic processes in the community assembly, microbial community structure tends to develop in a deterministic manner (Liang et al., 2020). Based on the functions of fungi predicted by using the FUNGuild database, we found that the AMF fungi community functional classification was significantly reduced under RR + N. AMF play an important role in ecosystem functions and regulate ecosystem stability by providing plants with N and P nutrients through symbiotic association with plant roots (Cotton et al., 2015; Yang et al., 2018). Previous studies found that N enrichment negatively affected AMF spore abundance, species richness, and biodiversity (Egerton-Warburton and Allen, 2000; Liu et al., 2013; Chen et al., 2017). A possible explanation for the significant decrease of AMF under RR + N treatment observed in the current study may be that N addition increased the inorganic nitrogen content (ammonia and nitrate nitrogen) of the soil. Plants prefer to utilize inorganic nitrogen in the soil for their own growth, and fertilization would reduce the demand for inorganic nitrogen produced by microbial decomposition and mineralization. Moreover, the AMF are a significant fungal community that participates in soil nitrogen mineralization (Cavagnaro et al., 2012; Bender et al., 2015). The reduction of soil water content also reduces the leaching of soil nutrient elements. Plants would require fewer nutrient elements from AMF community decomposition. Correspondently, the relative abundance of AMF was significantly reduced under RR + N conditions in our study.

The AMF of belowground ecosystem communities is mainly dominated by the phylum Glomeromycota (Johnson et al., 2013). We calculated the absolute abundance of the Glomeromycota through the absolute quantification of the ITS gene, and our results found that the absolute abundance of Glomeromycota did not significantly change between control and RR + N. However, it still exhibited a decreasing tendency, which was somewhat consistent with our fungal function prediction results. Our results further indicated that rainfall addition had positive effects on the phylum Glomeromycota. Rainfall addition increased the leaching of soil nutrients, especially NO_3_^–^. This would greatly increase plant demand for nitrogen, phosphorus, and other nutrients, and may cause AMF to symbiotically interact with plant roots to provide more of these nutrients to the host plants. Therefore, the absolute abundance of AMF significantly increased under rainfall addition. Mantel test showed that the rates of root exudate C from *A. polyrhizum* significantly affected the relative abundance of Glomeromycota. Because the Glomeromycota may have a symbiotic relationship with *A. polyrhizum* roots, the relative abundance of these fungi may be affected to a greater degree by plant root exudates. In addition, regarding the functional microbial groups involved in the soil carbon and nitrogen cycles, the response of AMF under the interaction of N and rainfall is more sensitive than that of other groups, suggesting that the interaction between these two factors has a significant impact on AMF over short timescales.

## Conclusion

In this study, we studied how the diversity and function of soil bacteria and fungi responded to the interaction between N addition and rainfall patterns in a temperate steppe of Inner Mongolia. The interaction between N addition and altered rainfall patterns had obvious effects on the diversity of these microbial communities. Additionally, the C:N ratios of root exudates from *A. polyrhizum* affected not only the diversity of soil bacteria, but also the diversity of fungi. The functions of bacteria did not significantly change, while the AMF in fungal function classification was obviously reduced under RR + N, suggesting that plants will first use the inorganic nitrogen in the soil for their growth, reducing the demand for inorganic nitrogen produced by AMF decomposition and mineralization. The absolute abundance of the phylum Glomeromycota significantly increased under the treatment of rainfall addition, potentially illustrating that AMF were more sensitive to changes in nitrogen and rainfall patterns. Our findings suggest that this may provide novel and important insights for understanding and predicting microbial-mediated ecological processes under global climate change. Lastly, our experiment examined the short-term response of bacterial and fungal diversity and functions to changes in soil nitrogen and moisture. However, there is still a need for further research about the long-term response mechanism of bacterial and fungal functions and plant–soil–microbe interactions.

## Data Availability Statement

The datasets presented in this study can be found in online repositories. The name of the repository and accession number(s) can be found below: National Center for Biotechnology Information (NCBI) BioProject, https://www.ncbi.nlm.nih.gov/bioproject/, PRJNA826987 and PRJNA826724.

## Author Contributions

CX designed this study. YY and LL conducted the study. YY, CX, JZ, SW, and YZ drafted the manuscript. All authors contributed to revisions and have read and approved the manuscript.

## Conflict of Interest

The authors declare that the research was conducted in the absence of any commercial or financial relationships that could be construed as a potential conflict of interest.

## Publisher’s Note

All claims expressed in this article are solely those of the authors and do not necessarily represent those of their affiliated organizations, or those of the publisher, the editors and the reviewers. Any product that may be evaluated in this article, or claim that may be made by its manufacturer, is not guaranteed or endorsed by the publisher.

## Footnotes

1
http://mem.rcees.ac.cn:8080/
