# Supplementary material for: The Diversity and Function of Soil Bacteria and Fungi Under Altered Nitrogen and Rainfall Patterns in a Temperate Steppe
Source: Front Microbiol. 2022 Jun 28;13:906818. doi: 10.3389/fmicb.2022.906818 (PMC9238322; doi:10.3389/fmicb.2022.906818)
Supplement: Supplementary file 1 [file Table_1.docx]

Table S1 The effects of nitrogen and rainfall and their interaction on the physical and chemical properties of soils and plants root exudation properties in August 2018. Different treatments: Con: Control; RR: Rainfall Reduction; RA: Rainfall Addition; N: Nitrogen Addition; RR+N: Rainfall Reduction and Nitrogen Addition; RA+N: Rainfall Addition and Nitrogen Addition. Values are means ± SE (n=5).

|  | Con | RR | RA | N | RR+N | RA+N |
| --- | --- | --- | --- | --- | --- | --- |
| Aboveground biomass (g m^2^) | 169.82±7.28 b c | 149.46±8.55 c | 192.91±9.76 a b | 191.21±11.96 a b | 173.50±7.82 b c | 219.14±10.47 a |
| Belowground biomass (g m^2^) | 785.95±146.49 | 747.89±138.30 | 891.32±185.10 | 891.88±173.62 | 762.18±134.50 | 1138.35±146.69 |
| Soil moisture (%) | 9.62±0.10 b | 9.00±0.14 c | 10.41±0.12 a | 9.62±0.10 b | 8.99±0.09 c | 10.58±0.05 a |
| SMBC (mg C kg^-1^) | 198.50±1.86 b | 187.70±2.41 c | 198.72±1.73 b | 206.10±2.87 a | 204.29±2.37 a b | 210.42±2.24 a |
| SMBN (mg C kg^-1^) | 19.66±0.21 d | 19.58±0.18 d | 20.17±0.26 c d | 21.01±0.33 a b | 20.43±0.20 b c | 21.38±0.22 a |
| SMBC: SMBN | 10.10±0.08 a | 9.59±0.14 b | 9.86±0.17 a b | 9.82±0.18 a b | 10.00±0.08 a b | 9.85±0.19 a b |
| NO_3_^-^ (mg kg^-1^) | 1.94±0.05 c d | 1.86±0.06 d | 2.22±0.05 c | 4.61±0.10 b | 4.52±0.15 b | 5.03±0.17 a |
| NH_4_^+^ (mg kg^-1^) | 7.40±0.25 b | 7.44±0.28 b | 7.88±0.19 b | 10.77±0.49 a | 10.76±0.21 a | 11.40±0.50 a |
| Stipa krylovii root C exudation rates (%) | 17.33±0.22 b c d | 17.46±0.03 a b c | 18.08±0.49 a b | 17.05±0.37 c d | 18.42±0.43 a | 16.42±0.15 d |
| Stipa krylovii root N exudation rates (%) | 2.12±0.06 b c | 2.60±0.05 a | 2.26±0.03 b | 1.92±0.06 d | 2.13±0.08 b c | 2.06±0.6 c d |
| Stipa krylovii root C: N exudation rates (%) | 7.61±0.20 b | 6.73±0.14 c | 8.31±0.17 a b | 8.34±0.43 a b | 8.99±0.41 a | 8.58±0.27 a |
| Allium polyrhizum root C exudation rates (%) | 15.02±0.16 e | 15.52±0.10 d | 14.75±0.09 e | 16.80±0.18 b | 17.41±0.09 a | 16.38±0.13 c |
| Allium polyrhizum root N exudation rates (%) | 2.90±0.06 b | 3.10±0.07 a | 2.67±0.04 c | 2.52±0.05 c | 2.61±0.04 c | 2.36±0.04 d |
| Allium polyrhizum root C: N exudation rates (%) | 5.18±0.15 b c | 5.01±0.13 c | 5.54±0.06 b | 6.69±0.20 a | 6.67±0.14 a | 6.96±0.13 a |

SMBC and SMBN represent soil microbial biomass carbon and nitrogen, respectively. Different letters represent significant difference (p < 0.05) according

to the Duncan’s post-hoc test.
